# Stretching Profile Correlated With the Aromatic Properties of Aged Light Cheese Enriched With Milk Globule Membrane Components

**DOI:** 10.1002/fsn3.70330

**Published:** 2025-05-25

**Authors:** Tulay Ozcan, Halil Riza Avci, Gokce Keser, Filiz Cavus

**Affiliations:** ^1^ Department of Food Engineering Bursa Uludag University Bursa Turkey; ^2^ Food and Feed Control Central Research Institute Bursa Turkey

**Keywords:** buttermilk, fat replacer, macrostructure, volatile compounds

## Abstract

This study used buttermilk‐rich milk fat globule membrane content as a fat substitute in reduced‐fat cheese samples. With the change in fat content, sensory and textural properties such as texture, flavor, and color, and technological changes in macrostructural, aromatic, and melting properties were comparatively investigated. In cheese samples, hardness and resistance to extension values increased depending on the buttermilk powder content. In sample L (7.5/100 g buttermilk powder), the hardness value increased by 178.04% and the resistance to extension value increased by 681.07% compared to the light control sample (L). However, this change did not affect the brittleness and melting quality. After a long‐term ripening process, changes occurred in the composition of volatile aroma compounds, and the compounds with the highest concentrations were determined as hexanoic, octanoic, acetic acid, 2,3‐butanone, 1‐butanol, 3‐methyl, 2‐hexadecanol, 2‐heptanol, 4‐heptanol, 2‐propenal, methional, pentanoic acid‐methyl ester, and acetic acid‐methyl ester. Acetic acid, acetic acid‐methyl ester, and 2‐propenal compounds were determined in higher amounts in buttermilk powder samples. In the L (7.5/100 g) sample, the acetic acid, 4‐heptanol, 2‐propenal, and acetic acid methyl ester amounts, respectively, increased by 20.21%, 19.36%, 217.71%, and 54.60% compared to the full‐fat control sample (F). While the panelists stated that the sensory overall acceptability was not affected by adding buttermilk powder, the L (7.5/100 g) sample was evaluated as more rigid and less aromatic than the other cheese varieties. In light of the data of this study, it was determined that buttermilk, an important dairy by‐product, can be used effectively in cheeses with reduced fat content. This study is valuable in terms of using sustainable additives and green transformation practices for the nutrition of future generations in recent years.

## Graphical abstract

In this study, milk fat globule membrane content was used as a fat substitute in aged light cheeses. It was determined that buttermilk can be used in reduced‐fat cheeses with its sensory and technological effects. The study shows the value of a sustainable additive for the nutrition of future generations.

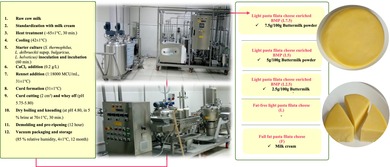

## 1 Introduction

Milk fat is a component that attracts attention with its nutritional value in milk, which affects the functional properties of the cheese as well as its technological parameters such as texture, aroma, and color. The fat content of the cheese affects the biochemical changes that occur during ripening and determines the characteristic properties and energy value of the product, depending on the cheese type (Feeney et al. 2021; Martin et al. 2024).

Reduced fat means that a product contains at least 25% less fat than the original version, meaning “reduced” refers to the amount of fat removed from the original product. By the way, low‐fat means a product contains 3 g of fat or less per serving, and 30% or less of the total calories per serving are from fat (Anonymous 2022). According to the Turkish Food Codex Regulation on Nutrition and Health Claims (2017) and European Commission (2012) No. 1047, foods that contain no more than 3 g (1.5 g for liquid foods) in 100 g solid food are “low (low) fat,” 100 g solid food. Foods in which the fat is not more than 0.5 g are defined as “fat‐free.”

Recently, due to the increasing number of nutrition‐related health problems such as obesity and cardiovascular diseases, consumers' demands for low‐fat dairy products and healthier food options are increasing (Gao et al. 2024). However, according to the Food and Agriculture Organization, a 22% increase in milk production is predicted by 2027. Since approximately one‐third of the global milk supply is used for cheese, cheese production worldwide is expected to increase further (Zhao et al. 2023). With approximately 1400 species, cheese represents the most diverse group among dairy products (Murtaza et al. 2024). Therefore, there is demand for low‐fat cheese due to consumer health concerns and changing lifestyle preferences for less fat, sugar, calories, and cholesterol (Caputo et al. 2023; Gao et al. 2024). However, despite the constant emphasis on dietary recommendations to reduce fat intake, consumers are reluctant to compromise on the taste and practicality of food (Zhang 2022).

In fatty cheeses, milk fat makes up about half of the total dry matter, thus increasing cheese yield. Reducing the fat content has negative effects on the sensory and physical properties of the cheese, while also causing economic losses due to slow ripening. The most common defects in fat‐free or low‐fat cheeses are increased cheese hardness and dispersibility, the formation of a rubber‐like off‐texture, the perception of foreign tastes and aromas, and the development of undesirable color defects (Kayihura 2024; Wen et al. 2021). To prevent and reduce defects in low‐fat cheeses, it is recommended to implement innovative technologies, appropriate starter culture/coculture selection, enzyme addition, structure regulators, fat substitutes, and emulsion systems (multiple and multilayer emulsions, etc.) (Aydinol and Ozcan 2018; Keser and Ozcan 2023; Qi et al. 2021).

Stretching and melting properties are important quality criteria in pasta filata cheeses. Reducing the fat content in boiled cheeses (the curd boiling process applied to cheeses) causes the cheese to have a harder texture and poorer melting and extension (extension). Optimal macro–micro‐structural design in low‐fat cheeses can be achieved by increasing the water content of the cheese. The protein concentration in cheese, and therefore the interaction and reformulations between proteins and other constituents, can be affected by the change in composition (Akarca et al. 2023; Aydinol and Ozcan 2018; Mohamed 2015).

Proteins are widely used in food products due to their properties, such as water absorption, gelation, and emulsion stabilization. In recent years, the green revolution and health‐promoting diets have increased the interest in proteins obtained from by‐products. The effectiveness of these components depends on factors such as molecular properties, pH, ionic strength, and the presence of polysaccharides Amiri et al. (2024). In recent years, protein‐based water‐soluble fat mimetics have enabled the reduction or elimination of fat in products where the fat is emulsified in water. Protein‐based fat replacers also change the flavor profile by binding flavor components (Aydinol and Ozcan, 2018; Patel et al. 2020; Amiri et al. 2024). Buttermilk has the potential to show this effect with its characteristic milk components, such as casein and whey proteins, in its structure. While 77%–81% of these proteins are casein, whey proteins are approximately 20%. It is reported that the phospholipid concentration in buttermilk, which contains milk proteins, lactose, vitamins, and minerals equivalent to skim milk, is higher than in skim milk (Hickey et al. 2017a; Bielecka et al. 2024). The liquid called “buttermilk,” obtained by clarifying or destabilizing milk cream, was considered a worthless by‐product of the dairy industry for many years. At this stage, the buttermilk containing the milk fat globule membrane (MFGM) is exposed at the bottom of the butter drum as the fat globules break down during churning. A special interest in its composition and therapeutic effects has emerged recently. The primary function of this membrane structure is to prevent milk fat molecules from coming together and protect them from external factors. This membrane, which passes into the buttermilk during the formation of butter, has been studied by nutritional scientists in recent years, and many food researchers have defined this membrane structure as a “functional molecule” (Bielecka et al. 2024; Ozcan et al. 2024; Sakkas et al. 2022; Szkolnicka et al. 2020). Considering the nutritional composition of buttermilk from butter production, the nutraceutical potential in the dairy industry makes this by‐product an important source for functional food production. Buttermilk contains high levels of characteristic milk components such as casein and whey proteins. It is a by‐product rich in phospholipid and bioactive peptide content and attracts attention with its emulsification effect in foods. In addition, these compounds positively affect health‐related cardiovascular diseases, the immune system, and cancer. The composition of buttermilk also enables the development of new and innovative therapeutic foods for the prevention of various chronic diseases with its cholesterol‐lowering, blood pressure‐lowering, and antioxidative stress‐reducing effects (Avci and Ozcan 2020; Ozcan and Demiray‐Teymuroglu 2020; Panou and Karabagias 2025).

Buttermilk contains MFGM, which is rich in phospholipids. MFGM is a protein–lipid biopolymer that originates from the apical surface of mammary epithelial cells and surrounds fat globules in milk (Manoni et al. 2020; Ozcan et al. 2024). These phospholipids are essential components of the human cell membrane and are the most important building blocks of the brain, nerve, muscle, heart, and liver tissues. The high phospholipid content of buttermilk makes this by‐product a novel techno‐functional ingredient for use in many food products such as bread, chocolate, snacks, margarine, meat, and dairy products (Pan et al. 2023; Panou and Karabagias 2025).

In this study, low‐fat pasta filata‐type cheese was produced. Buttermilk was used in varying amounts as a fat replacer to modify the changes in textural and sensory properties that may occur in cheese by reducing the amount of fat and the macrostructure and melting properties of cheese.

## 2 Materials and Methods

### 2.1 Materials and Reagents

The cow milk used to produce aged light cheese was supplied by Ozseymenler Food and Dairy Products Company in Bursa, Turkiye. The buttermilk powder was supplied by Enka Milk (Konya, Turkiye), the starter culture (CH‐351) by Maysa Food (Istanbul, Turkiye), and the rennet (Fermento 220) by Intermak (Konya, Turkey). The reagents H_2_SO_4_ (65%, d: 1.55 g/mL; CAS: 7664‐93‐9), C_5_H_12_O (d: 0.8104 g/mL; CAS: 123–51‐3), K_2_CrO_4_ (5.0%; CAS: 7789‐00‐6), HCl (0.1 N, CAS: 7647‐01‐0), NaOH (10 M, CAS: 1310‐73‐2), H_3_BO_3_ (4.0%, CAS: 10043–35‐3), and Kjeldahl tablets were received from a research laboratory and chemical company.

### 2.2 Method

#### 2.2.1 Aged Light Cheese Production

**FIGURE 1:**
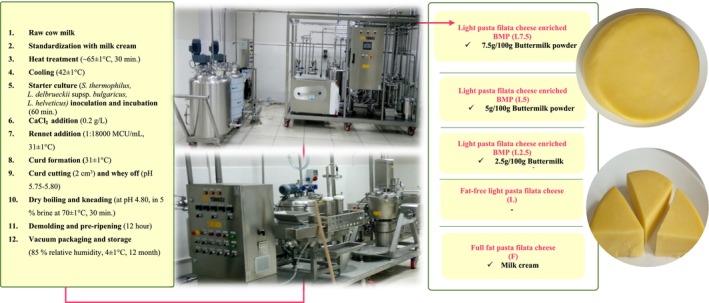
Flow chart of the aged light cheese production (BMP: buttermilk powder).

Different trial productions were conducted to determine the application procedures and buttermilk powder (BMP) ratios for production. According to these productions, the production procedures that gave the most appropriate textural and sensory results were optimized (data not shown). The BMP ratios were determined as 2.5/100, 5.0/100, and 7.5/100 g. The production procedure of cheese samples is given in Figure 1.

#### 2.2.2 Chemical Analysis

In cheese samples, dry matter (Gravimetric method; ISO 5534 2004), fat (Gerber method; ISO 1735 2004), and salt content (Mohr method; AOAC 2018) were determined.

#### 2.2.3 Proteolysis

In cheese samples, protein, in addition to determining the content of total nitrogen (TN) (Kjeldahl method, ISO 8968‐1 (2014)) and water‐soluble nitrogen (WSN) Kuchroo & Fox, (1982), was calculated using the formula ripening index [(WSN/TN) × 100] Ozcan and Kurdal (2012).

#### 2.2.4 Texture Profile Analysis

Texture parameters were measured using a TA‐XT plus texture analyzer (Stable Micro Systems, Surrey, UK). Hardness (g) and brittleness (mm) parameters were determined using an HDP/BS blade probe set conditioned at room temperature before analysis. Resistance to extension (g) and stretch quality (g/mm) parameters were measured with an A/CE (extensibility rig). Measurement conditions were determined as the HDP/BS attachment, (pretest speed 1.0 mm/s, test speed 2.0 mm/s, posttest speed 10.0 mm/s, and distance 10.0 mm), and the A/CE attachment with a PT 100 temperature sensor test speed 20.0 mm/s, posttest speed 20.0 mm/s, distance 270 mm, temperature 55°C, and samples of 60 g. Cheese samples stored at refrigerator temperature (4°C ± 1°C) were sliced (3 × 3 mm) and analyzed immediately. Results were recorded in a Texture Expert TE32 version 6.0 computer software (Biegalski et al. 2022; Li 2011).

#### 2.2.5 Volatile Aroma Component Analysis

The solid‐phase microextraction (SPME) method was used to extract the aroma compounds. Following the SPME, the injector was placed in the 5 g cheese sample. The samples were incubated for 30 min at a mixing speed of 4000 rpm at 40°C. After the extraction process was completed, the SPME injector was placed in the injection chamber of the GC and injected into the Agilent GC‐QTOF‐MS (USA). The results were compared with the SureMass scope and flavor classification in the device library, and quantification was expressed in percent. Parameters for GC‐QTOF were determined as follows: column start temperature 45°C, column‐type Agilent VF‐Wax ms (30 m × 0.25 mm × 0.25 μm), injection block temperature 280°C, transfer line temperature 250°C, ionization type EI (70 eV), mass analyzer‐type quadrupole‐time of flight, injection type splitless, injection volume 1 μL, carrier gas helium (1 mL/s), and analysis time 63 s (Plessas et al. 2011).

#### 2.2.6 Sensory Profile Analysis

For sensory analysis, consumers’ overripened samples were asked to indicate how much they liked or disliked each product on a 9‐point hedonic scale (9 = like extremely and 1 = dislike extremely) based on color, appearance, odor, flavor, salty, taste, body and texture, brittleness, hardness, stickiness, resilience, chewiness, and general acceptance. Moreover, in the sensory test, quantitative descriptive analysis (QDA) was performed by a trained panel of 25‐ to 55‐year‐olds (ISO 8586, 2012). Cheese samples (20°C) were randomly presented to the panelists with unsalted crackers and water. The panelists were asked to score the sample attributes' intensities (1–15). During the training sessions, they evaluated pasta filata cheeses to create a specific vocabulary for describing sensory attributes. The final list of descriptors used and their definitions are given in Table 1 (Biegalski and Cais‐Sokolińska 2023; Cuffia et al. 2019).

**TABLE 1:** Descriptive attributes and definitions used to evaluate aged light cheese.

Descriptor	Definition
Aromatic tastes	
Whey Powder	Odor/flavor arising from whey at room temperature
Creamy	Odor/flavor arising from creamy at room temperature
Free Fatty Acid	The taste is associated with hydrolyzed/oxidized fats
Moisty	Moisture is released by the product in the mouth during early mastication
Nutty	Flavors of various nuts, such as hazelnuts, almonds, or cashews, in cheese
Metallic	A metallic taste is often noted with exposure to metals or metal‐containing compounds
Basic Tastes	
Sour	The primary taste associated with citric acid and lactic acid
Bitter	Primary taste is produced by diluted aqueous solutions such as quinine and caffeine
Salty	Primary taste produced by aqueous solutions of sodium chloride
Umami	Fundamental taste elicited by certain peptides and nucleotides
Bite	The complex sensation accompanied by shrinking, drawing, or puckering of the mucous surface in the mouth
Fermented	Sour odor associated with natural yogurt
Textural Specifications	
Hardness	Resistance to a given deformation
Stickiness	The pastiness of the cheese against the palate and around the teeth during mastication
Brittleness	The extent to which cheese fragments are increasingly perceived during mastication
Dispersion	The extent to which cheese fragments are perceived during mastication
Firmness	Force required to compress between tongue and palate/resistance of cheese to crushing or deformation
Granular	Perception of course particles in the mouth
Chewiness	Number of chews required to masticate the sample to reduce it to a consistency suitable for swallowing
Gummy	The force required to remove the cheese sample that adheres to the mouth surface

#### 2.2.7 Data Analysis

One‐way analysis of variance (ANOVA) was conducted for all the results using Minitab 17 software (Minitab Inc., State College, PA, USA). When differences were significant (*p* < 0.05), the means were compared using Fisher's test. Results were given as mean ± standard deviation. Principal component analysis (PCA) and correlation were conducted using the OriginPro 2024 software package (OriginLab Corporation, USA). PCA and agglomerative hierarchical clustering (AHC) analysis were applied to the volatile metabolites detected using XLSTAT (Addinsoft, New York, USA).

## 3 Results and Discussion

### 3.1 Cheese Composition and Proteolysis Properties

The results of the composition of aged light cheese samples are given in Table 2. The samples' dry matter, fat, fat in dry matter, salt, and salt in dry matter amounts were found to be significant at the *p* < 0.05 level. It was determined that the amount of dry matter increased, and the amount of fat decreased with the increase in the ratio of BMP added to the samples. The fat in dry matter also decreased in proportion to the fat ratio. Although there was a slight difference between the samples in the amounts of salt and salt in dry matter, the amount of salt was higher in the L2.5 sample, and the amount of salt in dry matter was higher in the L and L2.5 samples (Table 2). Hickey et al. (2017a) investigated the effect of buttermilk water and BMP addition on Cheddar cheeses. They found that cheeses containing 10% BMP had higher moisture, salt, and salt content in dry matter. They determined that the amount of fat in dry matter decreased with the increase in BMP.

**TABLE 2:** Composition of aged light pasta filata cheese.

Samples	Dry matter (%)	Fat (%)	Fat in dry matter (%)	Salt (%)	Salt in dry matter (%)
F	52.13 ± 0.172^c^	23.70 ± 0.100^a^	46.64 ± 0.118^a^	1.58 ± 0.006^c^	3.20 ± 0.047^c^
L	46.65 ± 0.139^d^	9.60 ± 0.100^e^	20.56 ± 0.132^c^	1.54 ± 0.006^d^	3.51 ± 0.035^a^
L2.5	52.03 ± 0.156^c^	11.23 ± 0.058^c^	21.35 ± 0.170^b^	1.71 ± 0.010^a^	3.46 ± 0.035^a^
L5.0	52.77 ± 0.098^b^	11.47 ± 0.058^b^	21.58 ± 0.166^b^	1.64 ± 0.010^b^	3.27 ± 0.006^bc^
L7.5	55.82 ± 0.095^a^	10.60 ± 0.100^d^	18.74 ± 0.206^d^	1.66 ± 0.006^b^	3.34 ± 0.025^b^
*p*	**	**	**	**	**

*Note:* Different superscript (a–e), letters represent significant differences (*p* < 0.01) between aged light cheese samples. F: full‐fat cheese; L: light cheese; L2.5: 2.5/100 g BMP‐enriched cheese; L5.0: 5.0/100 g BMP‐enriched cheese; L7.5: 7.5/100 g BMP‐enriched cheese.

** *p* < 0.01.

The results of protein, WSN, TN, and ripening index, examined to determine the proteolysis properties of the samples, are given in Table 3. The TN, protein, WSN, and ripening index results in aged light cheese samples were significant at *p* < 0.05. It was determined that with the increase in BMP ratio, TN and protein increased, while the WSN and ripening index values decreased (Table 3). Researchers have determined that standardizing the milk to be processed into cheese with buttermilk or adding BMP during salting causes a decrease in protein levels. Govindasamy‐Lucey et al. (2006), Guinee and McSweeney (2006), and Hickey et al. (2017b) state that one of the reasons for this is related to the increase in moisture content, and that it occurs because the fat is retained less in the structure. However, according to the results of the study, there was an increase in the protein content of the samples. The use of BMP as a fat substitute and, therefore, the relative decrease in the fat content with the increase in BMP ratio caused the protein content to increase (Table 3). In cheeses, the ripening period generally affects the degree of proteolysis. As the cheese's storage period increases, the proteolysis degree also increases. Although the degree of proteolysis varies according to cheese type, it is stated that pasta filata‐type cheeses, such as Mozzarella, undergo limited proteolysis (Khattab et al. 2019). The amount of nitrogen that can be dissolved at pH 4.6 indicates primary proteolysis. It usually occurs by degrading −αs1 and −β caseins by nonmicrobial proteases (Cuffia et al. 2019). Hickey et al. (2017a) determined lower amounts of soluble nitrogen in Cheddar cheeses containing buttermilk than those without. As stated in previous studies, the lower levels of primary proteolysis in cheeses containing buttermilk are due to the interactions between denatured whey proteins, caseins in curd, and biocomponents in buttermilk.

**TABLE 3:** Proteolysis of aged light cheese.

Samples	Protein (%)	Water‐soluble nitrogen (%)	Ripening index	Total nitrogen (%)
F	27.96 ± 0.266^d^	0.73 ± 0.010^b^	16.82 ± 0.356^b^	4.39 ± 0.053^d^
L	28.40 ± 0.249^d^	0.79 ± 0.006^a^	17.79 ± 0.100^a^	4.43 ± 0.008^d^
L2.5	34.70 ± 0.268^b^	0.67 ± 0.010^c^	12.32 ± 0.136^c^	5.44 ± 0.013^b^
L5.0	33.69 ± 0.189^c^	0.57 ± 0.003^d^	10.71 ± 0.041^d^	5.33 ± 0.008^c^
L7.5	36.75 ± 0.164^a^	0.44 ± 0.013^e^	7.52 ± 0.244^e^	5.81 ± 0.018^a^
*p*	**	**	**	**

*Note:* Different superscript (a‐e) letters represent significant differences between aged light cheese samples. F: full‐fat cheese; L: light cheese; L2.5: 2.5/100 g BMP‐enriched cheese; L5.0: 5.0/100 g BMP‐enriched cheese; L7.5: 7.5/100 g BMP‐enriched cheese.

** *p* < 0.01.

### 3.2 Textural Properties

The results of the textural properties of low‐fat ripened cheese samples are given in Table 4. While the hardness values were determined at the highest level in the L5 and L7.5 samples, the highest resistance to extension value was determined in the L7.5 sample (*p* < 0.05). It was determined that the hardness values increased with the BMP ratio added to the samples. When the samples' brittleness and stretch quality parameters were examined, it was determined that adding BMP did not affect these properties (*p* > 0.05). A study by Tunick (2010) determined that Mozzarella cheeses with low‐fat content had high viscosity due to their higher protein content. The melting of the cheese is affected by the casein–casein, casein–water interactions, and the transition of the fat in the structure to the liquid state. The melting properties of cheeses also vary depending on the casein‐fat and water composition that occurs during storage and the proteolysis levels. The level of proteolysis may be due to casein hydration. In addition, the protein change in the soluble form at medium and high water content causes increased softness and easier reliability. The increase in textural hardness during storage makes it more difficult for casein to transition to a soluble form and affects the level of proteolytic degradation. As a result, elasticity and cohesiveness may change (Aydinol and Ozcan 2018; Bielecka et al. 2024). The density of the protein network in low‐fat cheese is significantly increased, reducing the moisture‐to‐protein ratio and ultimately resulting in a firm texture in low‐fat cheddar cheese (Zhang 2022). Similarly, in this study, buttermilk in cheese samples increased the amount of protein, and higher values were determined in the hardness and resistance to extension parameters in the cheese texture.

**TABLE 4:** Textural properties of aged light cheese.

Samples	Hardness (g)	Brittleness (mm)	Resistance to extension (g)	Stretch quality (g/mm)
F	2582.38 ± 383.860^bc^	8.00 ± 0.790^a^	25.22 ± 0.212^bc^	−0.02 ± 0.009^a^
L	1903.71 ± 29.520^c^	6.53 ± 1.028^a^	18.44 ± 1.131^c^	−0.01 ± 0.002^a^
L2.5	3259.77 ± 102.900^b^	6.07 ± 0.145^a^	52.67 ± 3.074^b^	−0.06 ± 0.079^a^
L5.0	5071.24 ± 583.444^a^	7.41 ± 1.330^a^	31.16 ± 7.498^bc^	−0.51 ± 0.900^a^
L7.5	5293.14 ± 150.490^a^	6.39 ± 0.150^a^	144.03 ± 27.166^a^	−0.49 ± 0.164^a^
*p*	**	ns	**	ns

*Note:* Different superscript (a‐e) letters represent significant differences between aged light cheese samples. F: Full‐fat cheese; L: Light cheese; L2.5: 2.5/100 g BMP‐enriched cheese; L5.0: 5.0/100 g BMP‐enriched cheese; and L7.5: 7.5/100 g BMP‐enriched cheese.

Abbreviation: ns, not significant.

** *p* < 0.01.

### 3.3 Volatile Aroma Compounds

The results of volatile aroma compounds of low‐fat aged cheese samples are given in Table 5. Thirty‐two volatile aroma compounds were detected, eight of which were acids, six were ketones, nine were alcohols, four were aldehydes, one was furan, and four were ester compounds. The compounds with the highest concentration in cheese samples were determined to be hexanoic, octanoic, acetic acid, 2,3‐butanone, 1‐butanol, 3‐methyl, 2‐hexadecanol, 2‐heptanol, 4‐heptanol, 2‐propenal, methional, pentanoic acid‐methyl ester, and acetic acid‐methyl ester.

**TABLE 5:** Volatile aroma compounds in aged light cheese samples.^+^

No.	Samples	F	L	L2.5	L5.0	L7.5
	Compounds					
	Acids					
1	Butanoic acid	2.01	1.36	2.2	2.68	1.98
2	Hexanoic acid	23.91	21.19	23.33	20.49	21.16
3	Heptanoic acid	0.76	0.27	0.15	0.2	0.29
4	Octanoic acid	7.69	7.14	6.23	6.7	6.8
5	Butanoic acid, 3‐methyl	0.58	0.55	0.52	0.48	0.4
6	Nonanoic acid	nd	1.35	1.57	2.81	2.95
7	Pentanoic acid	0.62	0.57	0.48	0.37	0.31
8	Acetic acid	11.68	10.24	14.48	13.79	14.04
	Ketones					
9	2‐Propanone	1.12	1.27	1.26	1.15	1.09
10	1‐Octen‐3‐one	1.12	0.97	0.83	0.79	0.65
11	2‐Nonanone	1.53	2.06	0.46	1.42	0.49
12	3‐Buten‐2‐one	1.14	0.95	1.02	0.93	0.9
13	2‐Heptanone	1.85	1.93	2.08	2.11	1.19
14	2,3‐Butanone	2.7	1.55	1.86	1.12	1.08
	Alcohols					
15	1‐Butanol, 3‐methyl	4.8	4.02	3.6	2.15	1.88
16	4‐Heptanol	5.2	3.61	2.85	1.66	1.88
17	2‐Hexadecanol	3.09	2.7	3.02	3.18	3.15
18	2‐Octanol	0.32	0.28	0.25	0.23	0.35
19	Benzene‐ethanol	0.95	0.82	0.94	0.65	nd
20	13‐Heptadecyn‐1‐ol	nd	0.52	0.8	2.77	1.19
21	1‐Hexanol	2.24	2.12	1.52	1.21	1.67
22	2‐Heptanol	9.75	13.08	11.01	10.76	9.46
23	2‐Hexadecanol	nd	nd	nd	nd	6.6
24	4‐Heptanol	5.32	6.45	6.49	6.58	6.35
	Aldehydes					
25	3‐Furaldehyde	1.24	nd	nd	nd	nd
26	2‐Propenal	1.75	4.12	5.74	6.32	5.56
27	2‐Butenal	1.12	nd	nd	nd	nd
28	Methional	1.05	4.12	3.46	3.52	3.35
	Furans					
29	2‐Vinylfuran	0.48	0.31	0.24	0.15	0.13
	Esters					
30	2‐Propenoic acid, methyl ester	1.96	2.19	1.75	1.35	0.52
31	Pentanoic acid, methyl ester	2.71	2.01	1.89	2.21	1.36
32	2‐Propenoic acid, 2‐methyl propyl ester	0.26	0.32	0.35	0.34	0.41
33	Acetic acid, methyl ester	1,74	2,05	1,65	2,71	2,69

*Note:* ^+^The concentration data of volatile compounds are shown as the mean value of peak volume. F: full‐fat cheese; L: light cheese; L2.5: 2.5/100 g BMP‐enriched cheese; L5.0: 5.0/100 g BMP‐enriched cheese; L7.5: 7.5/100 g BMP‐enriched cheese.

Abbreviation: nd, not detected.

Acids are important volatile aroma compounds among the compound groups determined in cheeses. Zheng et al. (2024), who examined the aroma changes during storage of cream cheeses, found that the amounts of acetic acid, benzoic acid, 2‐methylpropionic acid, 3‐methylbutanoic acid, decanoic acid, hexanoic acid, octanoic acid, nonanoic acid, (E)‐hept‐2‐enoic acid, and butanedioic acid changed during storage. Acid compounds are formed by lipid, protein, and glycolysis metabolism during the ripening of cheeses. While the amount of hexanoic acid was not much affected by the BMP added to the cheese samples, the amount of octanoic acid decreased with the addition of BMP (Table 5). Several short‐chain fatty acids (hexanoic, octanoic, etc.) are reported to contribute to cheese flavor and aroma. Hexanoic acid is responsible for sweaty, pungent, and rancid flavors, while octanoic acid has a goaty–waxy flavor. It was stated by Wang et al. (2022) that the formation of 2‐heptanone by β‐oxidation of octanoic acid is supported. Acetic acid, formed by glycolysis and proteolysis metabolism, gives cheeses a sour and vinegary taste. Hexanoic acid, octanoic acid, and nonanoic acid produced by lactose fermentation and lipid hydrolysis cause sweet, sweaty, and vinegary aromas (Zheng et al. 2021). In this study, the amount of acetic acid increased with the addition of BMP. A study investigating the effect of changes in fat content on aroma compounds determined differences in the volatile aroma compounds of light and full‐fat Cheddar cheese samples. In another study where BMP and buttermilk were used as additives in Cheddar cheeses, it was determined that the cheeses obtained with the addition of buttermilk had higher free fatty acid content and also contained volatile compounds related to the aromas of hexanoic acid, octanoic acid, and nonanoic acid, similar to our study (Hickey et al. 2018).

Ketones are generally important components that create an aroma in cheeses due to their low threshold values and unique flavors. In this study, six ketone compounds were determined (Table 5). Of these compounds, 2‐heptanone and 2,3‐butanone were determined in higher amounts than the others. 2‐Nonanone is an important volatile component in fermented dairy products and is derived from the β‐oxidation of fatty acids and subsequent decarboxylation of β‐ketoacids. It also gives the cheese a floral/fruity aroma (Dan et al. 2022). In this study, the amount of 2‐nonanone varied with the changes in the lipid content of the samples. 2‐Heptanone and 2,3‐butanone cause a creamy odor and are formed by the auto‐oxidation of lipids (Xie et al. 2022). The amount of 2,3‐butanone was detected at lower levels in samples containing 5% and 7.5% BMP than in full (F) and low‐fat (L) cheese samples (Table 5).

Esters are formed mainly by esterification of fatty acids and alcohols. They have a floral and fruity odor with a low threshold value, which can neutralize the unpleasant odor caused by excessive acids and make the cheese flavor milder. Lactones in cheese are released by the esterification of hydroxy fatty acids in triglycerides (Zheng et al. 2021). Increasing the amount of BMP in cheese samples caused a decrease in the amounts of 1‐butanol, 3‐methyl, 2‐heptanol, and methional. However, there was no significant change in the amounts of 2‐hexadecanol. In cheeses with buttermilk added, the amount of 2‐heptanol was similar to the low‐fat control sample and higher than the full‐fat control sample. The amount of 2‐propenal was higher in samples containing BMP than in F and L control samples. The amount of methional was determined to be lower than the low‐fat control sample and higher than the full‐fat control sample. In the L7.5 sample, the amount of pentanoic acid and methyl ester was lower than that of the other samples.

The addition of BMP caused an increase in the amount of acetic acid and methyl ester in the L5 and L7.5 samples (Table 5). The fat content of cheeses is one of the important milk components affecting the volatile aroma compounds. A study determined that low‐fat Feta cheeses contained higher aldehyde concentrations and lower alcohol, ketone, and ester compounds than full‐fat Feta cheeses (Andic et al. 2014). Salum et al. (2018) and Oluk (2023) identified 3‐methyl‐1‐butanol and 2‐heptanol compounds in cheeses. These compounds have fruity, alcoholic, green, balsamic, and woody odors (Kilcawley et al. 2018). 1‐Butanol‐3‐methyl is released during amino acid metabolism. In addition, it is stated that alcohol compounds tend to decrease during the ripening of cheeses, which may be due to the esterification reaction between alcohols and aldehydes (Zheng et al. 2024).

### 3.4 Sensory Properties

The sensory properties of aged light cheese samples were evaluated using the Hedonic Scale and QDA, and the results are given in Tables 6 and 7, respectively. When the hedonic scale evaluation results of the samples were examined, it was determined that all parameters were statistically significant (*p* < 0.01). Sensory evaluation scores were recorded above 7, and all samples were detected at a high sensory acceptability level (Table 6).

**TABLE 6:** Sensory–hedonic scale of aged light cheese.

Samples	Color	Appearance	Odor	Flavor	Salty	Taste	Body and texture	Brittleness	Hardness	Stickiness	Resilience	Chewiness	General acceptance
F	9.00^a^	9.00^a^	9.00^a^	8.88^c^	9.00^a^	8.75^b^	9.00^a^	9.00^a^	9.00^a^	9.00^a^	8.65^b^	8.98^a^	8.75^c^
L	8.88^b^	8.80^b^	8.70^b^	8.50^d^	8.50^c^	8.50^c^	8.50^b^	8.50^b^	8.50^b^	8.50^b^	8.23^c^	8.50^b^	8.50^d^
L2.5	9.00^a^	9.00^a^	9.00^a^	8.95^b^	9.00^a^	8.95^a^	8.98^a^	8.98^a^	9.00^a^	8.98^a^	8.68^b^	9.00^a^	8.95^b^
L5.0	9.00^a^	9.00^a^	9.00^a^	9.00^a^	9.00^a^	9.00^a^	9.00^a^	9.00^a^	9.00^a^	9.00^a^	8.73^a^	9.00^a^	9.00^a^
L7.5	8.28^c^	8.38^c^	8.50^c^	7.93^e^	8.75^b^	8.13^d^	7.58^c^	7.63^c^	7.63^c^	7.13^c^	7.38^d^	7.73^c^	7.68^e^
*p*	**	**	**	**	**	**	**	**	**	**	**	**	**

*Note:* Different superscript (a‐e) letters represent significant differences between aged light cheese samples. F: full‐fat cheese; L: light cheese; L2.5: 2.5/100 g BMP‐enriched cheese; L5.0: 5.0/100 g BMP‐enriched cheese; L7.5: 7.5/100 g BMP‐enriched cheese.

Abbreviation: ns, not significant.

** *p* < 0.01.

**TABLE 7:** Quantitative descriptive sensory analysis (QDA) of aged light cheese.

Types	F	L	L2.5	L5.0	L7.5	*p*
Aromatic Tastes						
Whey powder	0.00^b^	0.00^b^	0.00^b^	0.00^b^	0.25^a^	**
Creamy	0.00^b^	0.25^a^	0.00^b^	0.00^b^	0.00^b^	**
Free fatty acid	0.75^a^	0.50^b^	0.00^c^	0.00^c^	0.00^c^	**
Moisty	0.25^b^	0.75^a^	0.00^c^	0.00^c^	0.00^c^	**
Nutty	0.00^b^	0.25^a^	0.00^b^	0.00^b^	0.00^b^	**
Metallic	0.00^b^	0.25^a^	0.00^b^	0.00^b^	0.00^b^	**
Basic tastes						
Sour	5.25^e^	5.75^d^	6.38^c^	6.50^a^	6.43^b^	**
Bitter	0.50^b^	0.75^a^	0.00^c^	0.00^c^	0.00^c^	**
Salty	8.13^c^	7.75^d^	8.25^b^	8.25^b^	8.50^a^	**
Umami	0.00^b^	0.25^a^	0.00^b^	0.00^b^	0.00^b^	**
Bite	0.25^b^	0.50^a^	0.00^c^	0.00^c^	0.00^c^	**
Fermented	10.25^a^	9.25^e^	9.38^d^	9.50^b^	9.43^c^	**
Textural Specifications						
Hardness	8.00^d^	7.25^e^	8.50^c^	8.75^b^	11.25^a^	**
Stickiness	8.25^c^	8.75^a^	8.50^b^	8.50^b^	5.00^d^	**
Brittleness	4.50^c^	4.00^d^	4.75^b^	4.75^b^	9.75^a^	**
Dispersion	6.75^c^	6.25^d^	7.00^b^	7.00^b^	8.25^a^	**
Firmness	7.25^d^	6.75^e^	7.75^c^	8.00^b^	9.50^a^	**
Granular	1.75^b^	1.75^b^	1.75^b^	1.75^b^	3.25^a^	**
Chewability	7.50^b^	6.75^c^	7.75^b^	8.00^a^	8.00^a^	**
Gumminess	8.25^a^	8.75^a^	7.25^b^	7.75^b^	5.25^c^	**

*Note:* Different superscript ^(a‐e)^, letters represent significant differences between aged light cheese samples. F: full‐fat cheese; L: light cheese; L2.5: 2.5/100 g BMP‐enriched cheese; L5.0: 5.0/100 g BMP‐enriched cheese; L7.5: 7.5/100 g BMP‐enriched cheese.

Abbreviation: ns, not significant.

** *p* < 0.01.

The sensory perception intensities of aromatic taste, basic taste, and textural properties of aged light cheese samples were determined with QDA. It was determined that the samples containing buttermilk were perceived as saltier, and the fermented taste properties were perceived as higher (> 9). Adding buttermilk to the cheese samples caused higher differences, as can be understood from the scores in the textural properties of the products. It was determined that the samples' hardness, firmness, and brittleness scores increased with the buttermilk addition rate. The stickiness values of the samples decreased inversely proportional to these properties (Table 7).

**FIGURE 2:**
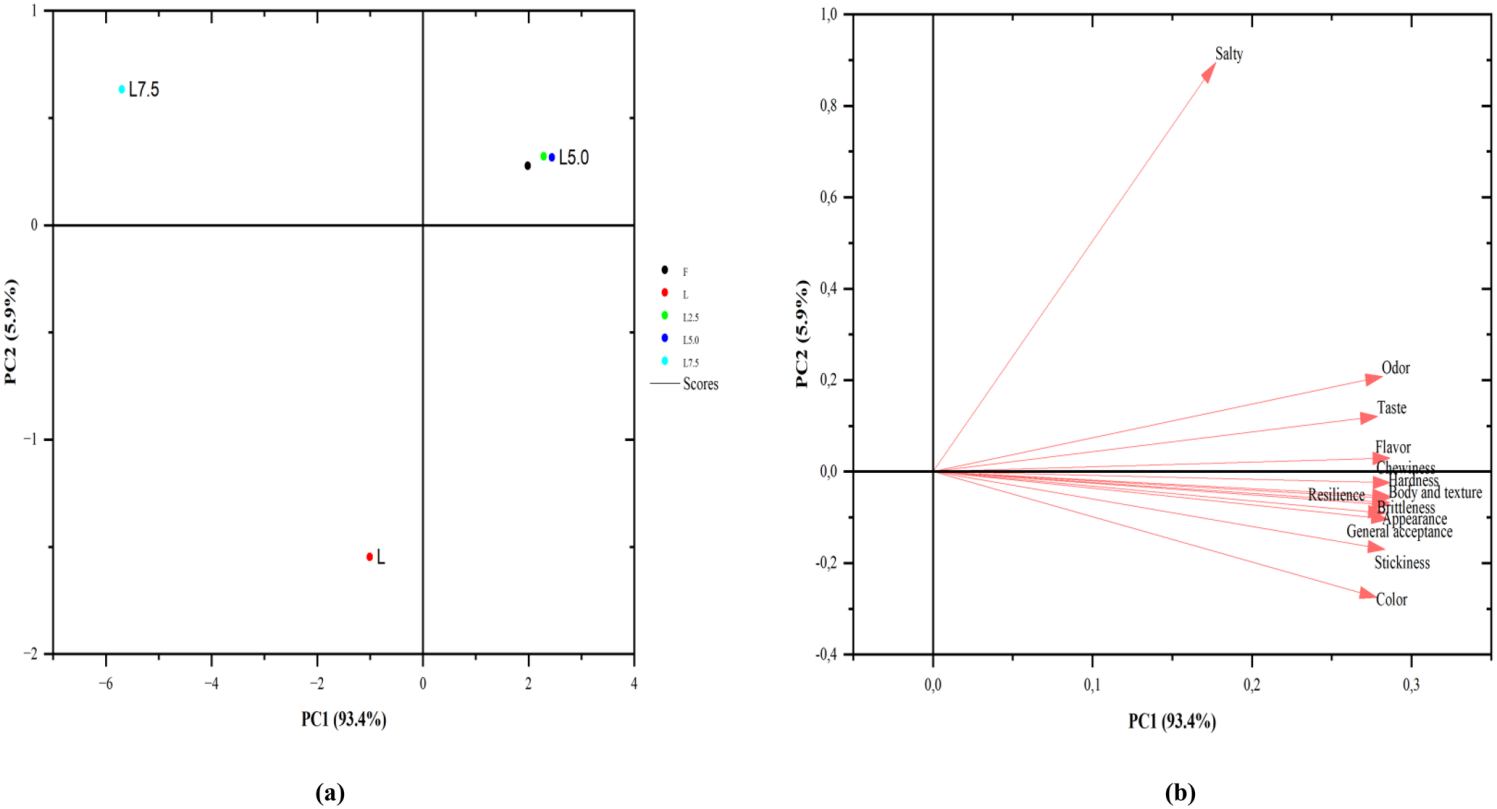
Plot of the PCs for the hedonic test (a: score plot, b: loading plot). Abbreviations: F: full‐fat cheese; L: light cheese, L2.5: 2.5/100 g BMP‐enriched cheese; L5: 5.0/100 g BMP‐enriched cheese; L7.5: 7.5/100 g BMP‐enriched cheese.

**FIGURE 3:**
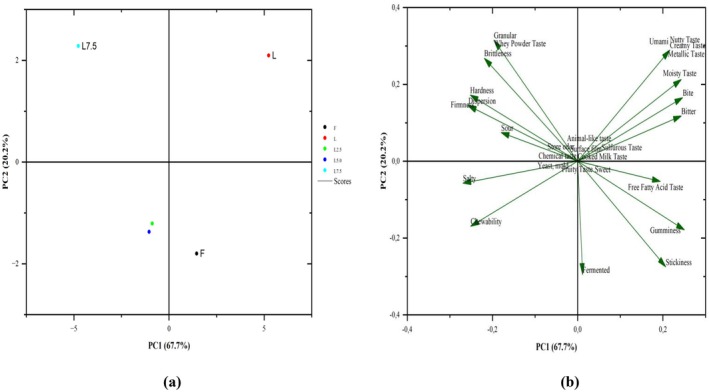
Plot of the PCs for the QDA (a: score plot, b: loading plot). Abbreviations: F: full‐fat cheese; L: light cheese; L2.5: 2.5/100 g BMP‐enriched cheese; L5: 5.0/100 g BMP‐enriched cheese; L7.5: 7.5/100 g BMP‐enriched cheese.

The results of hedonic and QDA parameters are given in Figures 2 and 3 by transferring them to PCA graphs. The sensory attributes of products were examined using PCA, and the maximum variance was obtained at 99.3%. With this PCA score, it can be concluded that overripened cheese samples are acceptable for consumption. Principal components PC1 and PC2 explained 93.4% and 5.9% of the variance, respectively (Figure 2a,b). According to the factor loadings of hedonic evaluation results, F, L5, and L2.5 samples were in the same group, and these samples were determined to be similar in terms of sensory evaluation profile. The features of L7.5 and L samples were found to be different by the panelists (Figure 2a). PC1 represented the total variance in the present study and included the variables with the highest weighting coefficients. The definitions (except salty) used in the hedonic evaluation can be explained by PC1 and similar factor loadings (Figure 2b).

The QDA parameters of aged light cheese samples were examined using PCA. Principal components PC1 and PC2 explained 67.7% and 20.2% of the variance, respectively. According to the factor loadings of the QDA evaluation results, it was determined that the L5 and L2.5 samples were not different from each other. Unlike the results defined in the hedonic scale, the full‐fat cheese sample was separated as a group (Figure 3a). In the products, chemical, yeast–mold, cooked milk, animal‐like, sweet, fruity, store odor, and sulfurous taste were not felt and, therefore, were shown in the center in the PCA loading plot graph (factor loadings were zero). The sensory evaluation correlated fermented taste with free fatty acid taste. While stickiness and gumminess scores showed positive changes, hardness, firmness, brightness, and granular properties showed negative correlations with these parameters (Figure 3b).

### 3.5 Interaction Between Sensorial Parameters (Hedonic Scale‐Scored) and Composition and Textural Properties

**FIGURE 4:**
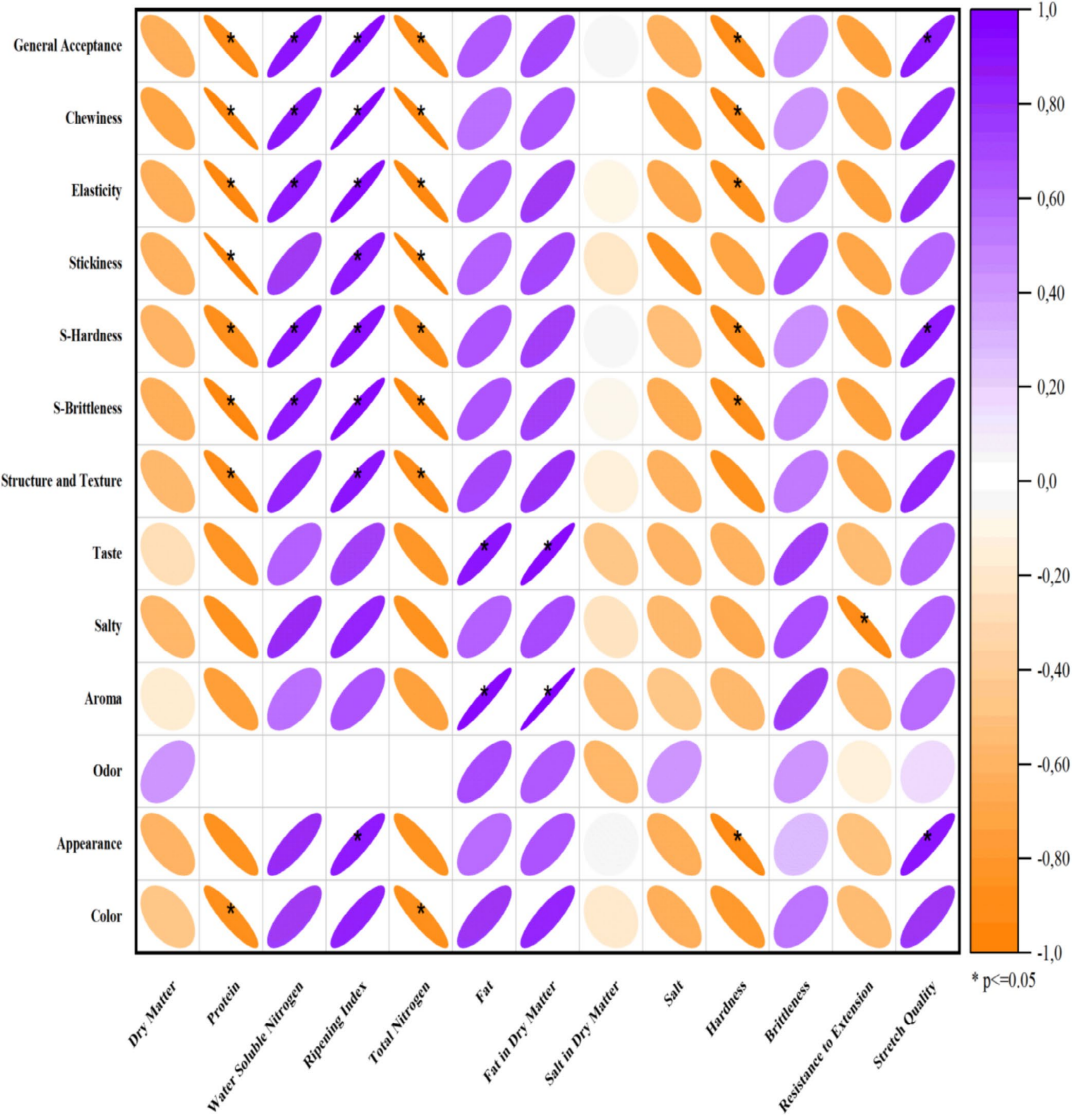
Correlation between sensorial parameters (hedonic scale‐scored) and composition, proteolysis, and textural properties of aged light cheese samples. *p < 0.05.

The correlation between the sensory properties and the changing composition, proteolysis, and textural properties of the samples with the addition of buttermilk is explained in Figure 4. A positive correlation was found between the samples' fat content, ripening index, and sensory properties. A negative correlation was found between resistance to extension, instrumental hardness, and TN content. During the sensory analysis, the panelists stated that the textural properties of the cheeses were generally an important parameter. It was explained that the increase in the dry matter content of the cheeses with high buttermilk content affected the resistance to extension values. This was reflected in the sensory acceptability of the samples.

### 3.6 PCA Analysis of QDA Parameters and Volatile Compounds

**FIGURE 5:**
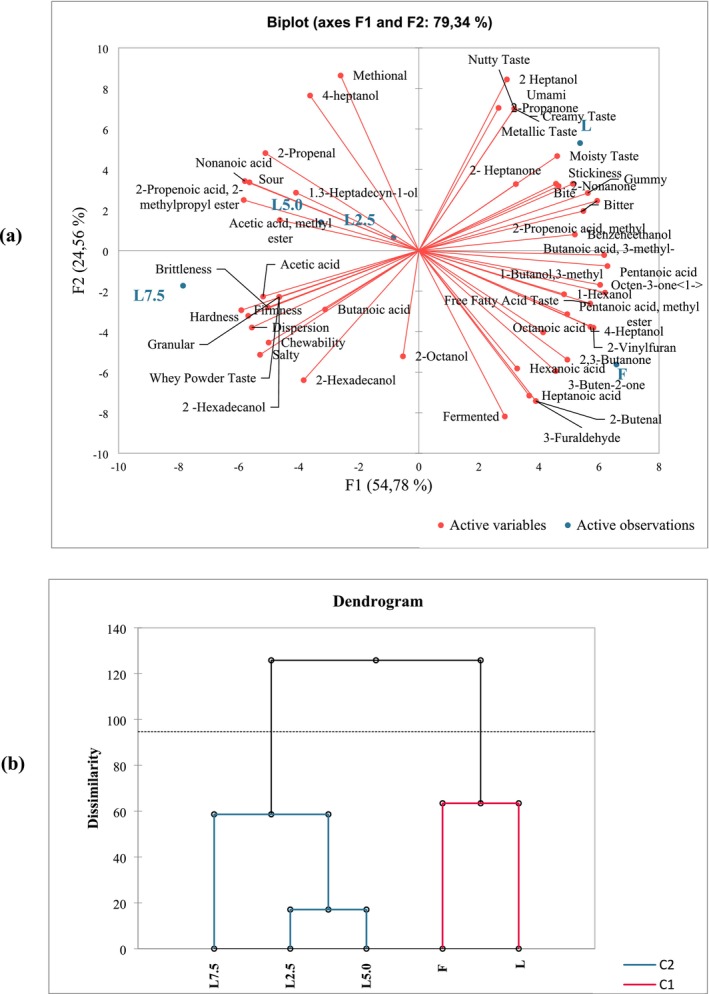
PCA (a) and AHC (b) analysis of QDA parameters and volatile compounds in aged light cheese samples. Abbreviations: F: full‐fat cheese; L: light cheese; L2.5: 2.5/100 g BMP‐enriched cheese; L5.0: 5.0/100 g BMP‐enriched cheese; L7.5: 7.5/100 g BMP‐enriched cheese.

In this study, buttermilk addition affected all detected properties of the samples to varying degrees. Chemometric analysis and chromatographic techniques provide a successful tool for discrimination between closely related samples Tarapoulouzi and Theocharis (2023). In general, aroma profiles in cheeses vary greatly with proteolysis and lipolysis reactions during ripening Keser & Ozcan, (2025). In this study, both PCA and AHC analyses were applied to the relative peak areas of all identified compounds of different cheese samples to find out the relationships between different amounts of buttermilk‐containing cheese samples and explore the similarities and differences between them. The graph of the PCA analysis performed using the volatile aroma compounds detected in aged light cheese samples, the QDA results, and the graph of the AHC analysis performed using the factors obtained as a result of this analysis are given in Figure 5. PCA output successfully reduced the number of variables into two components that explained 79.34% of the total variation of the dataset. PCA clustered buttermilk‐contained and uncontained cheese samples based on the presence and abundance of volatile aroma compounds and QDA scores. Different cheese samples could be divided into four main groups. Cheese samples were positioned remotely in the plot. The closeness of L2.5 and L5.0 was evident, but L, F, and L7.5 were segregated (Figure 5a). The AHC dendrogram (Figure 5b) categorized the cheese samples into two main clusters. Clusters I and II displayed F, L, L2.5, L5.0, and L7.5, respectively.

## 4 Conclusion

Recently, sustainable nutrition has attracted more attention due to the increase in the world population and the increasing interest in climate change issues. This study revealed a strong correlation between biochemical parameters, aromatic degradation, and sensory properties in cheese samples. Thirty‐two VOCs, including eight acids, six ketones, ten alcohols, four aldehydes, six esters, and one furan, were identified in aged light cheese samples, depending on proteolytic and lipolytic changes. While these results help understand the flavor changes in overripened cheese varieties, they also offer changing perspectives on developing innovative, aromatic, and healthy products containing buttermilk. Sustainable nutrition is a global nutrition model for access to safe and sufficient food. To reduce the carbon footprint in nutrition, evaluating food industry waste and biodiversity, clean water, and environmental and human health are important issues today. In this sense, using dairy industry wastes as functional additives will provide potential benefits.

## Author Contributions

**Tulay Ozcan:** investigation (equal), methodology (equal), project administration (equal), supervision (equal), validation (equal), writing – original draft (equal), writing – review and editing (equal). **Halil Riza Avci:** data curation (equal), formal analysis (equal), investigation (equal), project administration (equal). **Gokce Keser:** conceptualization (equal), formal analysis (equal), software (equal), writing – original draft (equal), writing – review and editing (equal). **Filiz Cavus:** conceptualization (equal), formal analysis (equal).

## Conflicts of Interest

The authors declare no conflicts of interest.

## Data Availability

Data will be made available on request.

## References

[fsn370330-bib-0001] Akarca, G. , A. Atik , I. Atik , and A. J. Denizkara . 2023. “A Comparison Study on Functional and Textural Properties of Mozzarella Cheeses Made From Bovine and Buffalo Milks Using Different Starter Cultures.” International Dairy Journal 141: 105622.

[fsn370330-bib-0002] Amiri, M. , S. E. Hosseini , G. Asadi , B. Khayambashi , and A. Abedinia . 2024. “Optimization of Microalgae Protein Extraction From *Scenedesmus obliquus* and Investigating Its Functional Properties.” Lebensmittel‐Wissenschaft & Technologie 198: 116028.

[fsn370330-bib-0003] Andic, S. , Y. Tuncturk , and G. Boran . 2014. “Changes in Volatile Compounds of Cheese.” In Processing and Impact on Active Components in Food, edited by V. Preedy , 231–239. Academic Press.

[fsn370330-bib-0004] Anonymous . 2022. “CFR—Code of Federal Regulations Title 21.” https://www.accessdata.fda.gov/scripts/cdrh/cfdocs/cfcfr/cfrsearch.cfm?.

[fsn370330-bib-0005] AOAC . 2018. “AOAC Official Method 960.29. Salt in Cheese.” AOAC International.

[fsn370330-bib-0006] Avci, H. R. , and T. Ozcan . 2020. “The Characterisation of Dairy Waste Buttermilk From Different Butter Processing Procedures.” Fresenius Environmental Bulletin 7: 5472–5478.

[fsn370330-bib-0007] Aydinol, P. , and T. Ozcan . 2018. “Production of Reduced‐Fat Labneh Cheese With Inulin and β‐Glucan Fibre‐Based Fat Replacer.” International Journal of Dairy Technology 71: 362–371.

[fsn370330-bib-0008] Biegalski, J. , and D. Cais‐Sokolińska . 2023. “Production of Sensorily Acceptable Pasta Filata Cheese With Partial Substitution of Sheep's Milk Powder in Different Forms.” Food 12: 1766. 10.3390/foods12091766 PMC10177789 37174304

[fsn370330-bib-0009] Biegalski, J. , D. Cais‐Sokolińska , and J. Wawrzyniak . 2022. “Effect of Packaging and Portioning on the Dynamics of Water–Fat Serum Release From Fresh Pasta Filata Soft Cheese.” Food 11: 296. 10.3390/foods11030296 PMC8834549 35159448

[fsn370330-bib-0010] Bielecka, M. M. , A. Florczuk , and M. Aljewicz . 2024. “An Evaluation of the Impact of Curdlan and Buttermilk Addition on the Functional Properties and Sensory Quality of Processed Cheese Analogs.” Molecules 30, no. 1: 66. 39795123 10.3390/molecules30010066 PMC11721369

[fsn370330-bib-0011] Caputo, V. , G. Sogari , and V. E. J. Loo . 2023. “Do Plant‐Based and Blend Meat Alternatives Taste Like Meat? A Combined Sensory and Choice Experiment Study.” Applied Economic Perspectives and Policy 45, no. 1: 86–105.

[fsn370330-bib-0012] Cuffia, F. , G. George , L. Godoy , G. Vinderola , J. Reinheimer , and P. Burns . 2019. “In Vivo Study of the Immunomodulatory Capacity and the Impact of Probiotic Strains on Physicochemical and Sensory Characteristics: Case of Pasta Filata Soft Cheeses.” Food Research International 125: 108606. 31554130 10.1016/j.foodres.2019.108606

[fsn370330-bib-0013] Dan, T. , H. Hu , T. Li , A. Dai , B. He , and Y. Wang . 2022. “Screening of Mixed‐Species Starter Cultures for Increasing Flavour During Fermentation of Milk.” International Dairy Journal 135: 105473.

[fsn370330-bib-0015] European Commission . 2012. “Commission Regulation (EU) No 1047/2012 of 8 November 2012 Amending Regulation (EC) No 1924/2006 With Regard to the List of Nutrition Claims Text With EEA Relevance.” Official Journal of the European Union.

[fsn370330-bib-0016] Feeney, E. L. , P. Lamichhane , and J. J. Sheehan . 2021. “The Cheese Matrix: Understanding the Impact of Cheese Structure on Aspects of Cardiovascular Health A Food Science and a Human Nutrition Perspective.” International Journal of Dairy Technology 74: 656–670.

[fsn370330-bib-0017] Gao, Y. , Y. Zhao , Y. Yao , et al. 2024. “Recent Trends in Design of Healthier Fat Replacers: Type, Replacement Mechanism, Sensory Evaluation Method and Consumer Acceptance.” Food Chemistry 447: 138982. 38489876 10.1016/j.foodchem.2024.138982

[fsn370330-bib-0018] Govindasamy‐Lucey, S. , T. Lin , J. J. Jaeggi , M. E. Johnsoni , and J. A. Lucey . 2006. “Influence of Condensed Sweet Cream Buttermilk on the Manufacture, Yield, and Functionality of Pizza Cheese.” Journal of Dairy Science 89: 454–467. 16428615 10.3168/jds.S0022-0302(06)72109-9

[fsn370330-bib-0019] Guinee, T. P. , and P. L. H. McSweeney . 2006. “Significance of Milk Fat in Cheese.” In Advanced Dairy Chemistry Volume 2 Lipids, edited by P. F. Fox and P. L. H. McSweeney , 377–440. Springer.

[fsn370330-bib-0022] Hickey, C. D. M. G. , J. O'Sullivan , D. Davis , et al. 2018. “The Effect of Buttermilk or Buttermilk Powder Addition on Functionality, Textural, Sensory and Volatile Characteristics of Cheddar‐Style Cheese.” Food Research International 103: 468–477. 29389637 10.1016/j.foodres.2017.09.081

[fsn370330-bib-0021] Hickey, C. D. , B. W. K. Diehl , M. Nuzzo , A. Millqvist‐Feurby , M. G. Wilkinson , and J. J. Sheehan . 2017a. “Influence of Buttermilk Powder or Buttermilk Addition on Phospholipid Content, Chemical and Bio‐Chemical Composition and Bacterial Viability in Cheddar Style‐Cheese.” Food Research International 102: 748–758. 29196008 10.1016/j.foodres.2017.09.067

[fsn370330-bib-0020] Hickey, C. D. , M. A. E. Auty , M. G. Wilkinson , and J. J. Sheehan . 2017b. “Influence of Process Temperature and Salting Methods on Starter and NSLAB Growth and Enzymatic Activity During the Ripening of Cheeses Produced With *Streptococcus thermophilus* and *Lactobacillus helveticus* .” International Dairy Journal 69: 9–18.

[fsn370330-bib-0024] ISO 1735 . 2004. “Cheese and Processed Cheese Products—Determination of Fat Content—Gravimetric Method (Reference Method).” IDF 5:2004; International Organization for Standardization: Geneva, Switzerland.

[fsn370330-bib-0025] ISO 5534 . 2004. “Cheese and Processed Cheese—Determination of the Total Solids Content (Reference Method).” IDF 4:2004; International Organization for Standardization: Geneva, Switzerland.

[fsn370330-bib-0059] ISO 8586 . 2012. Sensory Analysis‐Selection and Training of Sensory Assessors. International Organization for Standardization.

[fsn370330-bib-0026] ISO 8968‐1 . 2014. “Milk and Milk Products—Determination of Nitrogen Content—Part 1: Kjeldahl Principle and Crude Protein Calculation.” IDF 20–1:2014; International Organization for Standardization: Geneva, Switzerland.

[fsn370330-bib-0027] Kayihura, J. F. 2024. “Partitioning of Casein and Fat in Cheddar Cheese Manufacturing as Affected by Cheese Milk Standardisation: A Review.” International Journal of Dairy Technology 77: 35–49.

[fsn370330-bib-0058] Keser, G. , and T. Ozcan . 2025. “Cross‐Over Fermentation Dynamics and Proteomic Properties of Acid Gels with Indigenous *Lactobacillus* spp. Isolated From Cheeses.” Food Microbiology: 128. 10.1016/j.fm.2024.104700 39952741

[fsn370330-bib-0028] Keser, R. A. , and T. Ozcan . 2023. “Food Emulsions and Microstructural Design.” In Advanced Strategies for Agriculture‐2, edited by T. Beycioglu , 15–44. Iksad Publishing, Ankara, Turkiye.

[fsn370330-bib-0029] Khattab, A. R. , H. A. Guirguis , S. M. Tawfik , and M. A. Farag . 2019. “Cheese Ripening: A Review on Modern Technologies Towards Flavor Enhancement, Process Acceleration and Improved Quality Assessment.” Trends in Food Science and Technology 88: 343–360.

[fsn370330-bib-0030] Kilcawley, K. , H. Faulkner , H. Clarke , M. O'Sullivan , and J. Kerry . 2018. “Factors Influencing the Flavour of Bovine Milk and Cheese From Grass Based Versus Nongrass Based Milk Production Systems.” Food 7: 1–43. 10.3390/foods7030037 PMC5867552 29534042

[fsn370330-bib-0060] Kuchroo, C. N. N. , and P. F. Fox , 1982. “Soluble nitrogen in Cheddar cheese: Comparison of extraction procedures.” Milchwissenschaft 37, no. 6: 331–335.

[fsn370330-bib-0031] Li, X. M. 2011. “Correlation Analysis Between Measured Values of the Texture Analyzer and Scale Values of Sensory Evaluation for Food Hardness.” Advanced Materials Research 183: 882–886.

[fsn370330-bib-0032] Manoni, M. , C. Di Lorenzo , M. Ottoboni , M. Tretola , and L. Pinotti . 2020. “Comparative Proteomics of Milk Fat Globule Membrane (MFGM) Proteome Across Species and Lactation Stages and the Potentials of MFGM Fractions in Infant Formula Preparation.” Food 9: 1251. 10.3390/foods9091251 PMC7555516 32906730

[fsn370330-bib-0033] Martin, C. , M. Harel‐Oger , G. Garric , and S. Marette . 2024. “Impact of Sensory Properties and Their Appreciation on Willingness to Pay for Innovative Cheeses With Health Benefits.” Food Quality and Preference 118: 105207.

[fsn370330-bib-0034] Mohamed, A. G. 2015. “Low‐Fat Cheese: A Modern Demand.” International Journal of Dairy Science 10: 249–265.

[fsn370330-bib-0035] Murtaza, M. S. , A. Sameen , A. Rehman , et al. 2024. “Physicochemical, Techno‐Functional, and Proteolytic Effects of Various Hydrocolloids as Fat Replacers in Low‐Fat Cheddar Cheese.” Frontiers in Sustainable Food Systems 8: 1440310.

[fsn370330-bib-0036] Oluk, A. C. 2023. “Effect of Production Variations on the Composition, Textural and Microstructural Properties, and Volatile Compounds of Turkish White Cheese During Ripening.” Lebensmittel‐Wissenschaft & Technologie 173: 114348.

[fsn370330-bib-0038] Ozcan, T. , and E. Kurdal . 2012. “The Effects of Using a Starter Culture, Lipase, and Protease Enzymes on Ripening of Mihalic Cheese.” International Journal of Dairy Technology 65: 585–593.

[fsn370330-bib-0037] Ozcan, T. , and M. Demiray‐Teymuroglu . 2020. “Bioactive Components of Milk Fat Globule Membrane and Technological Applications.” International Journal of Scientific and Technological Research 6: 10–28.

[fsn370330-bib-0039] Ozcan, T. , L. Yilmaz‐Ersan , and N. Dinkci . 2024. “Technological and Health Effects of Butter By‐Products Rich in Phospholipids in Consumers’ Sustainable Diets.” In Consumer Perceptions and Food, 639–657. Springer Nature Singapore.

[fsn370330-bib-0040] Pan, J. , M. Chen , N. Li , et al. 2023. “Bioactive Functions of Lipids in the Milk Fat Globule Membrane: A Comprehensive Review.” Food 12: 3755. 10.3390/foods12203755 PMC10606317 37893646

[fsn370330-bib-0041] Panou, A. , and I. K. Karabagias . 2025. “Composition, Properties, and Beneficial Effects of Functional Beverages on Human Health.” Beverages 11, no. 2: 40.

[fsn370330-bib-0042] Patel, A. R. , R. A. Nicholson , and A. G. Marangoni . 2020. “Applications of Fat Mimetics for the Replacement of Saturated and Hydrogenated Fat in Food Products.” Current Opinion in Food Science 33: 61–68.

[fsn370330-bib-0043] Plessas, S. , A. Alexopoulos , A. Bekatorou , I. Mantzourani , A. A. Koutinas , and E. Bezirtzoglou . 2011. “Examination of Freshness Degradation of Sourdough Bread Made With Kefir Through Monitoring the Aroma Volatile Composition During Storage.” Food Chemistry 124: 627–633.

[fsn370330-bib-0044] Qi, W. , T. Li , Z. Zhang , and T. Wu . 2021. “Preparation and Characterization of Oleogel‐In‐Water Pickering Emulsions Stabilized by Cellulose Nanocrystals.” Food Hydrocolloids 110: 106206.

[fsn370330-bib-0045] Sakkas, L. , V. Evageliou , P. E. Igoumenidis , and G. Moatsou . 2022. “Properties of Sweet Buttermilk Released From the Churning of Cream Separated From Sheep or Cow Milk or Sheep Cheese Whey: Effect of Heat Treatment and Storage of Cream.” Food 11: 465. 10.3390/foods11030465 PMC8833928 35159618

[fsn370330-bib-0046] Salum, P. , G. Gokce , P. Kendirci , D. Bas , and Z. Erbay . 2018. “Composition, Proteolysis, Lipolysis, Volatile Compound Profile and Sensory Characteristics of Ripened White Cheeses Manufactured in Different Geographical Regions of Turkey.” International Dairy Journal 87: 26–36.

[fsn370330-bib-0047] Szkolnicka, K. , I. Dmytrów , and A. Mituniewicz‐Małek . 2020. “Buttermilk Ice Cream. New Method for Buttermilk Utilization.” Food Science & Nutrition 8: 1461–1470. 32180955 10.1002/fsn3.1429 PMC7063380

[fsn370330-bib-0048] Tarapoulouzi, M. , and C. R. Theocharis . 2023. “Discrimination of Anari Cheese Samples in Comparison With Halloumi Cheese Samples Regarding the Origin of the Species by FTIR Measurements and Chemometrics.” Analytica 4: 374–384.

[fsn370330-bib-0049] Tunick, M. H. 2010. “Activation Energy Measurements in Rheological Analysis of Cheese.” International Dairy Journal 20: 680–685.

[fsn370330-bib-0061] Turkish Food Codex Nutrition and Health Claims Regulation . 2017. “Ministry of Agriculture and Forestry, Communiqué No: 29960.” Turkiye.

[fsn370330-bib-0050] Wang, H. , Y. Li , X. Xia , Q. Liu , F. Sun , and B. Kong . 2022. “Flavor Formation From Hydrolysis of Pork Meat Protein Extract by the Protease From *Staphylococcus carnosus* Isolated From Harbin Dry Sausage.” LWT ‐ Food Science and Technology 163: 113525.

[fsn370330-bib-0051] Wen, P. , Y. Zhu , J. Luo , et al. 2021. “Effect of Anthocyanin‐Absorbed Whey Protein Microgels on Physicochemical and Textural Properties of Reduced‐Fat Cheddar Cheese.” Journal of Dairy Science 104: 228–242. 33189294 10.3168/jds.2020-18450

[fsn370330-bib-0052] Xie, Q. , B. Xu , Y. Xu , et al. 2022. “Effects of Different Thermal Treatment Temperatures on Volatile Flavor Compounds of Water‐Boiled Salted Duck After Packaging.” LWT ‐ Food Science and Technology 154: 112625.

[fsn370330-bib-0054] Zhang, D. 2022. “Improving Quality and Functionality of Low‐Fat Mozzarella Cheese Using Whey Proteins and Plant Proteins.” HKU Theses Online (HKUTO).

[fsn370330-bib-0055] Zhao, Y. , H. Khalesi , J. He , and Y. Fang . 2023. “Application of Different Hydrocolloids as Fat Replacer in Low‐Fat Dairy Products: Ice Cream, Yogurt and Cheese.” Food Hydrocolloids 138: 108493.

[fsn370330-bib-0056] Zheng, A. R. , C. K. Wei , M. S. Wang , N. Ju , and M. Fan . 2024. “Characterization of the Key Flavor Compounds in Cream Cheese by GC‐MS, GC‐IMS, Sensory Analysis and Multivariable Statistics.” Current Research in Food Science 8: 100772. 38840807 10.1016/j.crfs.2024.100772 PMC11150910

[fsn370330-bib-0057] Zheng, X. , X. Shi , and B. Wang . 2021. “A Review on the General Cheese Processing Technology, Flavor Biochemical Pathways and the Influence of Yeasts in Cheese.” Frontiers in Microbiology 12: 703284. 34394049 10.3389/fmicb.2021.703284 PMC8358398

